# Implementation and evaluation of the three action teaching model with learning plan guidance in preventive medicine course

**DOI:** 10.3389/fpsyg.2024.1508432

**Published:** 2024-11-07

**Authors:** Cai-Yun Chen, Ta-La Shi, Ruo-Yu Wang, Ning Li, Yi-Han Hao, Jia-Le Zhang, Ming Tang, Sha Liu, Guo-min Qin, Wei Mi

**Affiliations:** ^1^School of Public Health, Binzhou Medical University, Yantai, China; ^2^School of Health Management, Binzhou Medical University, Yantai, China; ^3^School of Stomatology, Binzhou Medical University, Yantai, China; ^4^The First School of Clinical Medicine, Binzhou Medical University, Yantai, China; ^5^School of Traditional Chinese Medicine, Binzhou Medical University, Yantai, China; ^6^Academic Affairs Office, Binzhou Medical University, Yantai, China

**Keywords:** learning plan guidance, three action teaching model, preventive medicine, teaching design, implementation and evaluation

## Abstract

**Background:**

Toward the close of the 20th century, Chinese scholars introduced a novel pedagogical approach to education in China, distinguished by its divergence from conventional teaching methods. This instructional strategy assumes a pivotal role in imparting indispensable medical knowledge to students within a meticulously structured and all-encompassing framework.

**Objective:**

The objective of this study is to assess the effectiveness of a novel teaching approach that integrates the three action teaching model with learning plan guidance within a preventive medicine course. Through this investigation, empirical evidence will be provided regarding the impact of utilizing learning guided by the three action teaching model with learning plan guidance as an innovative instructional method, thereby shedding light on its potential to enhance students’ autonomous learning in the field of preventive medicine.

**Methods:**

The control group consisted of 48 students from Class 2 of clinical medicine in grade 2021, who were taught using the traditional classroom teaching mode. Meanwhile, Class 1 served as the experimental group comprising 47 individuals, who received instruction through the three-action teaching model with learning plan guidance. Evaluation was conducted using course tests and questionnaires, and data analysis was performed utilizing t-tests, analysis of variance, and rank sum tests in SPSS software.

**Results:**

The average total score of the test group (79.44 ± 10.13) was significantly higher than that of the control group (70.00 ± 13.57) (*t* = 3.943, *p* < 0.001). Moreover, there were more experimental groups with total scores ranging from 80 to 89 and 90 to 100 compared to the control group (*Z* = 5.324, *p* = 0.002). The Subjective Evaluation System (SES) indicated that the experimental group (69.11 ± 8.39) outperformed the control group (61.23 ± 6.59) in terms of total scores (*t* = 5.095, *p* < 0.001), demonstrating superior performance in learning methods, emotions, engagement, and performance metrics (*p* < 0.05). Specifically, analysis using the Biggs study process questionnaire revealed that the experimental group exhibited higher levels of deep learning (*t* = 6.100, *p* < 0.001) and lower levels of superficial learning (*t* = −3.783, *p* < 0.001) when compared to the control group.

**Conclusion:**

The implementation of a novel teaching approach that integrates the three-action teaching model with learning plan guidance significantly enhances students’ academic achievements and fosters their intrinsic motivation for learning. The success of this pedagogical method can be attributed to the enhanced classroom efficiency exhibited by teachers as well as the heightened enthusiasm for learning displayed by students.

## 1 Introduction

Health professional education is central to achieving universal health coverage and the Sustainable Development Goals, and contributes significantly to health outcomes and public trust in health workers (Hassan et al., 2024). As a fundamental component of health medicine education, the complex nature of preventive medicine often presents great challenges to students. Consequently, enhancing the pedagogical effectiveness of preventive medicine and improving the knowledge proficiency of medical students have become focal points in educational reform initiatives (Liu et al., 2023).

Traditionally, preventive medicine education has predominantly followed conventional pedagogical approaches characterized by an intensive didactic methodology throughout the course (Zhang et al., 2024). In the traditional teaching mode, teachers focus on “teaching,” pay attention to whether students have mastered the teaching content, and mainly follow the one-way method, while ignoring the internalization and application of students’ knowledge (Yu et al., 2021). In the context of conventional pedagogical approaches, the methodologies are constrained by temporal and spatial limitations, resulting in diminished student engagement during class sessions (Kong et al., 2024). Moreover, prolonged exposure to such methods may elicit a sense of ennui among learners, which subsequentially diminishes the efficacy of instruction and manifests as suboptimal learning outcomes (Ilkiw et al., 2017). However, this approach often leads to a decline in self-directed learning capabilities and a lack of enthusiasm for knowledge acquisition among students. Additionally, the diverse educational backgrounds and varying learning competencies of students further complicate instructors’ ability to monitor individual progress and efficiently manage instructional tasks, thus hindering the achievement of optimal educational outcomes (Zhang et al., 2018; Xu et al., 2021). Optimizing classroom time and fostering students’ learning capabilities are essential avenues for educators to investigate within the framework of the ‘Double Reduction’ policy, representing a critical step toward improving educational quality in this context (Zhou, 2023). The progression of curriculum reform, combined with the dynamic advancements of the era, requires a shift from passive learning models toward more autonomous and engaged learning practices. Failure to adapt to these changes jeopardizes students’ preparedness or even marginalization in their subsequent professional pursuits (Zhang, 2023).

Currently, the learning guide has been extensively utilized in various university education settings and across multiple subject areas in teaching practice. The model underscores the primacy of students’ activities within the classroom, fostering the autonomous construction of conceptual frameworks. The acquisition of knowledge transcends mere impartation and passive reception; it entails active engagement by students in cognitive endeavors to initially formulate concepts, while also providing them with the opportunity to articulate their personal perspectives. The model encourages active communication and dialog among classmates and with instructors, thereby facilitating a profound comprehension of new concepts and nurturing the development of students’ creative thinking. This pedagogical approach starkly contrasts with traditional teaching methods, where knowledge is imparted in a linear fashion, focusing on the cultivation of students’ observational and practical skills (Huang, 2022; Xiao, 2023). Drawing upon over a year of empirical experience, educators at Zibo Middle School in Shandong Province, China have advocated for the implementation of a pedagogical strategy known as “learning plan guidance,” which emphasizes student-centered and developmental approaches. The process of its implementation primarily involves the following steps: establishing clear goals through the use of study plans; guiding learning through case-based instruction tailored to individual self-study situations; organizing discussions and attempting to resolve difficulties based on feedback from challenging information; teacher elaboration followed by student summarization; integration through linking related concepts to form a cohesive network; standardized training during class sessions for knowledge transfer and expansion (Jin, 2014). The innovative model promotes independent exploration and active engagement of students in the subject matter, guided by teachers. This facilitates a shift from passive absorption of knowledge to active discovery and mastery of scientific inquiry methods. In addition, it was emphasized cooperative learning between teachers and students, and puts an end to the former phenomenon of teachers’ “filling the classroom” and attaches importance to students’ active knowledge construction (Guo, 2024). The teaching method employed here is evidently distinct from the conventional approach, as it not only enhances teachers’ classroom efficiency but also fosters students’ learning autonomy, thereby augmenting overall learning outcomes (Chen, 2022).

The aim of this study is to investigate the implementation and effects of “learning plan guidance” in the field of preventive medicine, specifically focusing on its integration into blended learning environments. The objective is to enhance course content and classroom dynamics, improve students’ interest in learning, and ultimately contribute to elevating teaching standards for preventive medicine courses while enhancing students’ autonomous learning ability.

## 2 Materials and methods

### 2.1 Study subjects

In this quasi-experimental study, the pre-teaching learning of the class of 2021 clinical medicine professional 1 and 2 classes was analyzed through diagnostic assessment. Participants in this study had a maximum age of 23 and a minimum age of 19. There were no statistically significant differences in gender, learning interest attitude, and previous academic performance between the two groups (*p* > 0.05). The subjects for this study were selected based on their similarity in student population, male to female ratio, course-related academic performance, and learning attitude. The results indicated no statistically significant difference (*p* > 0.05) between the experimental group and the control group in these aspects. The control group, Class 2, comprised of 48 students and was instructed using the conventional classroom teaching approach. In contrast, the experimental group, Class 1, consisted of 47 students and implemented an innovative teaching methodology. This method involved structured learning guided by predefined learning plans and executed through a three-phase teaching strategy. This teaching research did not require ethical issues, but all participants signed written informed consent forms after being fully informed about the study details.

### 2.2 Study methods

The present study employed a comparative approach by categorizing participants into two distinct groups in order to assess the efficacy of different teaching methodologies. The control group adhered to a conventional pedagogical framework, wherein the instructional process unfolded through a series of structured phases. Initially, the educator devised a lesson plan that aligned with the curriculum requirements and created an accompanying PowerPoint presentation (PPT). Each teaching session comprised a 30-min lecture, utilizing the prepared PPT for content delivery, followed by a 10-min discussion session aimed at addressing queries and reinforcing the covered material. After the classroom interaction, homework assignments were distributed and subsequently reviewed by the educator, who provided personalized feedback to enhance student learning. The cycle culminated with the educator reflecting on class outcomes, extracting insights to refine and optimize future educational strategies. This represents the fundamental process of traditional teaching employed by the control group as well as a component of the novel instructional approach adopted by the experimental group.

The experimental group in the study participated in a structured learning model, which was systematically implemented in three distinct phases and supported by meticulously crafted learning plans. These plans were executed through a tripartite teaching methodology. Initially, this process commences with educators conducting an in-depth analysis and prognosis of the students’ learning contexts, leading to a diagnostic evaluation. Based on these insights, educators tailor learning plans for individual students. The preparatory phase plays a pivotal role in laying the foundation for the subsequent phase of self-directed learning, during which students are guided by a comprehensive study plan. This entails conducting an extensive review and preview of pertinent subject knowledge, identifying key areas of knowledge, and summarizing individual learning inquiries that serve to clarify responses to teachers’ questions.

Subsequently, the model progresses into an interactive phase. Among them, the discussion link is the core link of the implementation of the teaching mode of learning guide (Ye, 2023). Students are organized into small clusters to actively participate in collaborative discussions, critically analyze and share insights on the identified problems, as well as reflect on their independent learning experiences. These discussions culminate in the creation of group learning reports that not only document unresolved issues but also consolidate collective self-study experiences, providing a valuable resource for teacher evaluation.

**Figure 1:**
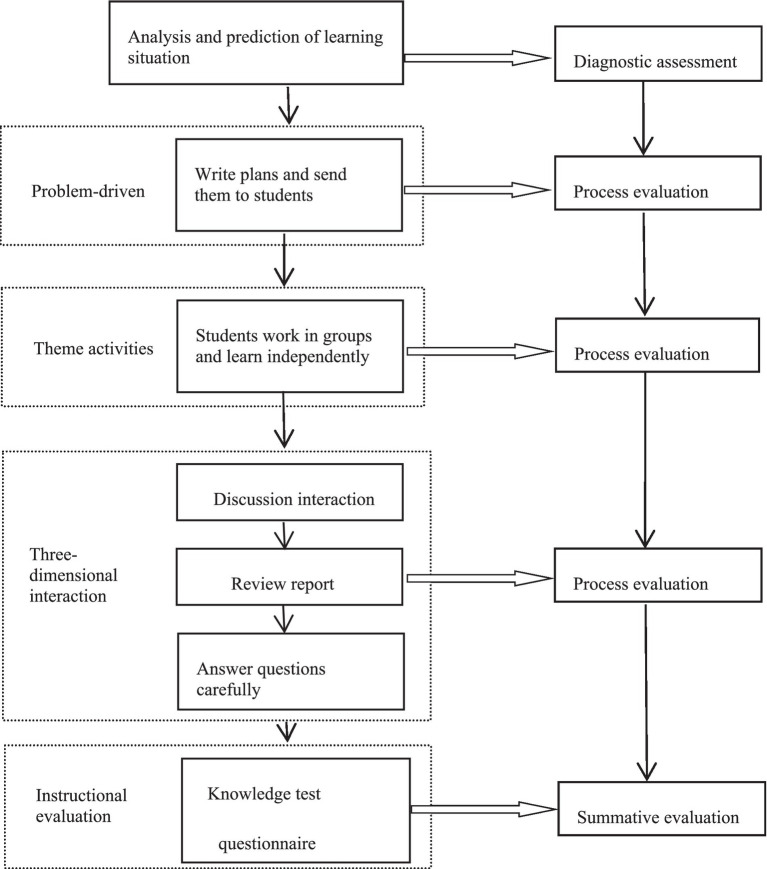
Implementation flow chart of the teaching mode of “the three action teaching model with learning plan guidance”.

The final phase centers on classroom interactions, wherein educators address inquiries by leveraging insights derived from group discussions and reports. This segment is crucial for reinforcing the learning objectives and elucidating any knowledge gaps that students may have encountered during their learning process. The effectiveness of this comprehensive teaching and learning process is assessed through a dual-faceted evaluation strategy that analyzes the substance and depth of group discussions and reports, while also evaluating student performance in a post-lesson examination. The comprehensive approach ensures a dynamic, interactive, and effective learning environment that is tailored to meet the diverse needs and abilities of students, thereby optimizing the educational outcome. The teaching mode is illustrated in Figure 1.

This mode emphasizes student-centered, situational creation and active construction of knowledge. Guided question chains are designed to stimulate students’ interest in inquiry, encourage students to explore knowledge in real or simulated situations through independent learning and group cooperation, and form their own understanding and application. Teachers should be facilitators and supporters of learning, providing diverse learning resources and timely feedback to promote students’ reflection and self-evaluation. Under the guidance of constructivism theory, the study plan guide aims to cultivate students’ critical thinking, innovation ability and independent learning ability, and realize the deep construction and transfer of knowledge.

### 2.3 Curriculum test

Upon completion of the course, formative assessments were conducted for both the experimental and control groups, utilizing an identical grading system framework. This grading system comprised three primary components: a final examination accounting for 50% of the overall grade, class examinations contributing 30%, and regular coursework assessments comprising the remaining 20%. This structured approach facilitated a consistent and comparative evaluation of student performance across both groups.

### 2.4 Questionnaire survey

The Curriculum Engagement Assessment Questionnaire is utilized to evaluate students’ active involvement in the course. The Student Engagement Scale (SES) is employed to assess students’ learning approach, emotional state, level of engagement, and academic performance. Participants are required to rate their responses on a five-point scale ranging from 1 (“completely dissimilar to me”) to 5 (“very similar”). The consistency of the SES in evaluating student participation is substantiated by its high reliability, as indicated by a pre-questionnaire Cronbach’s alpha coefficient of 0.830. Additionally, the presurvey showed good validity with a Kaiser–Meyer–Olkin (KMO) value of 0.770 and Bartlet’s test of sphericity *p* < 0.01. The Biggs questionnaire serves as a crucial assessment tool in delineating students’ engagement with deep and surface learning styles, encompassing their learning strategies and motivational levels. This instrument utilizes a structured scoring spectrum ranging from “1 = never” to “5 = always,” providing nuanced insights into student preferences and behaviors related to the learning process. Furthermore, its reliability is underscored by a robust Cronbach’s alpha coefficient of 0.802, indicating a high level of internal consistency among the questionnaire items. This statistical measure reassures educators and researchers about the instrument’s dependability in capturing the multifaceted aspects of students’ learning processes. The questionnaire demonstrates good reliability and validity.

The other SES scale is utilized to assess the feasibility of teachers in teaching and management, encompassing dimensions such as student management, implementation of teaching strategies, and classroom management. Participants provided responses on a five-point scale ranging from 1 (not at all) to 5 (completely able). The reliability of the SES was demonstrated by a pre-questionnaire Cronbach’s alpha coefficient of 0.776, indicating its suitability for evaluating teachers in terms of their teaching and management abilities. The pre-survey yielded a Kaiser–Meyer–Olkin (KMO) value of 0.414 and Bartlet’s test of sphericity with *p* < 0.01.

### 2.5 Statistical analysis

The statistical analyses were conducted using SPSS 25.0 software. The measured data were presented as mean ± standard deviation ( $\bar{x}$ *± s*), and the performance of the two sample groups was assessed using *t*-test, or rank sum test. With a test level *ɑ* = 0.05.

## 3 Results

The investigation comprised of two groups: the control group, comprising 48 cases, and the experimental group, encompassing 47 cases. Notably, the experimental group exhibited superior performance compared to the control group, thereby indicating the efficacy of the novel teaching methodology implemented by the experimental group.

### 3.1 Comparison of the test scores

As outlined in Table 1, when comparing the mean experimental score (*p* < 0.05) with that of the control group (92.52 ± 2.99), the experimental group exhibited a slightly lower mean score (90.46 ± 5.67) (*t* = −2.273, *p* = 0.025). Furthermore, the average final score for the experimental group was 73.37 ± 10.85, while it was 64.25 ± 11.20 for the control group (*t* = 4.136, *p* < 0.001), thereby emphasizing notable differences between both groups across various evaluation measures.

**Table 1:** Comparison of usual scores, experimental scores and final scores between the control group and the test group ( $\bar{x}$ *± s*).

Groupings	Number of people	Usual scores	Experimental scores	Final scores
Control group	48	92.15 ± 8.29	92.52 ± 2.99	64.25 ± 11.20
Test group	47	94.08 ± 4.89	90.46 ± 5.67	73.37 ± 10.85
*t*		1.416	−2.273	4.136
*P*		0.160	0.025	<0.001

It is evident from Supplementary Figure S1 and Supplementary Table S2 that the experimental group achieved a higher average total score (79.44 ± 10.13) compared to the control group’s average total score of (70.00 ± 13.57) (*t* = 3.943, *p* < 0.001). Moreover, there were more participants in the experimental group who scored within ranges of 80–89 and 90–100 than in the control group (*Z* = 5.324, *p* = 0.002).

### 3.2 Results of the students’ SES questionnaire

The mean total score (69.11 ± 8.39) of the experimental group was significantly higher than that of the control group (61.23 ± 6.59) (*t* = 5.095, *p* < 0.05), as presented in Supplementary Table S1. Supplementary Table S1 demonstrates statistically significant differences between the experimental and control groups across four key dimensions: learning method, learning emotion, learning participation, and learning achievement (*p* < 0.05).

As shown in Table 2, in terms of learning methods, the experimental group exhibited higher scores in pre-class preparation (3.70 ± 0.81) and conscientious study in class (4.02 ± 0.68), compared to the control group’s scores (2.40 ± 0.87) (*t* = 7.596, *p* < 0.001) for pre-class preparation and (3.42 ± 0.77) (*t* = 4.079, *p* < 0.001) for conscientious study in class. In terms of learning emotion, the control group scored lower (3.58 ± 0.85) than the experimental group (3.91 ± 0.75) (*t* = 2.023, *p* < 0.05). Regarding learning participation, active group discussion in the experimental group yielded a higher score (3.79 ± 0.78) compared to that of the control group (3.27 ± 0.96) (*t* = 2.873, *p* < 0.05). The scores for frequent discussion (3.85 ± 0.66) and assisting other students (3.53 ± 0.86) in the experimental group were significantly higher than those of the control group (3.08 ± 1.05) (*t* = 4.283, *p* < 0.001), as well as helping other students (2.94 ± 0.91) (*t* = 3.280, *p* = 0.001). Furthermore, all differences in terms of academic performance were statistically significant at a significance level of *p* < 0.05. The score for the experimental group with good test performance (3.83 ± 0.79) was significantly higher than that of the control group (3.15 ± 0.80) (*t* = 4.198, *p* < 0.001), and similarly, the score for the experimental group with high scores (3.70 ± 0.75) was significantly higher than that of the control group (3.25 ± 0.70) (*t* = 3.041, *p* < 0.05).

**Table 2:** Results of the SES questionnaire for students ( $\bar{x}$ *± s*).

Investigate the project	Experimental group	Control group	*t*	*p*
Learning methods				
Preview before class	3.70 ± 0.81	2.40 ± 0.87	7.596	<0.001
Study carefully in class	4.02 ± 0.68	3.42 ± 0.77	4.079	<0.001
Review after class	3.21 ± 0.81	3.08 ± 1.05	0.674	0.502
Take good notes	3.74 ± 0.79	3.44 ± 0.90	1.767	0.081
Learn emotions				
Do your best	3.98 ± 0.71	3.75 ± 0.89	1.391	0.168
Theory is linked to practice	3.91 ± 0.75	3.58 ± 0.85	2.023	0.046
What you learn is put into practice	3.64 ± 0.82	3.40 ± 0.89	1.378	0.171
Stay interested in the course	3.70 ± 0.88	3.52 ± 0.95	0.966	0.337
Really eager to learn	3.36 ± 1.01	2.98 ± 0.93	1.918	0.058
Learn to engage				
The group discussion was lively	3.79 ± 0.78	3.27 ± 0.96	2.873	0.005
Participate in online chats	3.06 ± 1.03	3.23 ± 0.97	−0.804	0.423
Frequent discussion of speeches	3.85 ± 0.66	3.08 ± 1.05	4.283	<0.001
Help other students	3.53 ± 0.86	2.94 ± 0.91	3.280	0.001
Academic performance				
The quiz performed well	3.83 ± 0.79	3.15 ± 0.80	4.198	<0.001
High score	3.70 ± 0.75	3.25 ± 0.70	3.041	0.003

### 3.3 Results of the Biggs questionnaire

The total deep learning score of the experimental group (28.94 ± 2.57) was significantly higher than that of the control group (25.83 ± 2.38) (*t* = 6.100, *p* < 0.001), as indicated in Supplementary Table S1. The total shallow learning score of the experimental group (25.26 ± 2.98) was found to be significantly lower than that of the control group (27.63 ± 3.07) (*t* = −3.783, *p* < 0.001). Additionally, a significant difference was observed in shallow learning motivation scores between the experimental group (12.70 ± 2.01) and the control group (13.98 ± 1.96), with lower scores recorded for the former (*t* = −3.134, *p* < 0.05). Similarly, a significant difference was noted in shallow learning strategy scores between both groups, with lower scores observed for the experimental group (12.57 ± 1.82) compared to those of the control group (13.65 ± 1.85) (*t* = −2.852, *p* < 0.05).

In Table 3, in terms of deep learning motivation, the experimental group had a higher satisfaction with learning (3.89 ± 0.67) than the control group (2.73 ± 0.87) (*t* = 7.336, *p* < 0.001). The experimental group had a higher score (3.77 ± 0.63) than the control group (3.13 ± 0.76) (*t* = 4.457, *p* < 0.001), this shows that the students in the experimental group are more willing to think deeply. In terms of deep learning strategies, the scores of the experimental group (3.72 ± 0.65) were higher than those of the control group (3.25 ± 0.73) (*t* = 3.339, *p* = 0.001). In terms of shallow learning motivation, the scores of the experimental group on doing as little as possible (3.19 ± 0.90) and not making an effort to understand the key points (2.81 ± 0.85) were lower than those of the control group on doing as little as possible if you think the course is boring (3.71 ± 0.90) (*t* = −2.801, *p* < 0.05) and no effort to understand key points (3.46 ± 0.85) (*t* = −3.725, *p* < 0.001). In terms of shallow learning strategies, the scores of students in the experimental group regarding there is no need to do anything except study (2.91 ± 0.72) and the best way to pass the exam is to memorize the main points of the exam (2.93 ± 0.71) are lower than those of students in the control group regarding there is no need to do anything except study (3.33 ± 0.83) (*t* = −2.624, *p* < 0.05) and the best way to pass the test is to memorize the score of the test points (3.38 ± 0.96) (*t* = −2.534, *p* < 0.05).

**Table 3:** Results of the Biggs questionnaire for students ( $\bar{x}$ *± s*).

Investigate the project	Experimental group	Control group	*t*	*p*
Deep learning motivation				
There is a deep sense of satisfaction in learning	3.89 ± 0.67	2.73 ± 0.87	7.336	<0.001
It’s fun to dive into any problem as well	3.77 ± 0.63	3.13 ± 0.76	4.457	<0.001
Study hard because the learning content is interesting	3.36 ± 0.70	3.04 ± 0.99	1.814	0.073
In most of the lessons, questions are answered	3.38 ± 0.77	3.04 ± 0.77	2.162	0.033
Deep learning strategies				
It takes a lot of work to get the answer	3.72 ± 0.65	3.25 ± 0.73	3.339	0.001
Often, take extra time to gain more knowledge	3.68 ± 0.73	3.42 ± 0.77	1.724	0.088
Keep learning and figuring out the key issues	3.51 ± 0.72	3.63 ± 0.91	−0.677	0.500
Spend a lot of time researching and discussing issues outside of class	3.62 ± 0.77	3.60 ± 0.76	0.082	0.935
Shallow learning motivation				
Spend the least amount of effort to pass this course	3.43 ± 0.80	3.48 ± 0.92	−0.302	0.763
If you do not find the course interesting, try to do as little as possible	3.19 ± 0.90	3.71 ± 0.90	−2.801	0.006
You do not need to work hard to understand just the key points	2.81 ± 0.85	3.46 ± 0.85	−3.725	<0.001
It is not necessary to read the contents of the examination without examination	3.28 ± 0.77	3.33 ± 0.83	−0.344	0.732
Shallow learning strategies				
Only those who are required by the syllabus should study carefully	3.53 ± 0.91	3.56 ± 0.77	−0.178	0.859
Rote memorization of knowledge points that you do not understand	3.21 ± 0.69	3.38 ± 0.84	−1.029	0.306
There is no need to do anything outside of studying	2.91 ± 0.72	3.33 ± 0.83	−2.624	0.010
The best way to pass the exam is to memorize the test points	2.93 ± 0.71	3.38 ± 0.96	−2.534	0.013

### 3.4 Results of the TSES questionnaire

As shown in Table 4, this TSES questionnaire is about teacher management and teaching feasibility. This questionnaire mainly includes three aspects: student management, the implementation of teaching and classroom management. Specifically, in student management, teachers in the experimental group communicated more with students and scored higher on helping students think critically (4.08 ± 0.51) than the control group (3.23 ± 0.44) (*t* = 4.469, *p* < 0.001), and the students in the experimental group were able to think independently, and their scores in cultivating students’ creativity (4.23 ± 0.60) were significantly higher than those in the control group (3.15 ± 0.69) (*t* = 4.254, *p* < 0.05). In terms of the implementation of teaching strategies, compared with the control group’s scores on measures of students’ understanding of the content taught (3.46 ± 0.52), designing good questions for students (2.92 ± 0.64), and higher level challenges faced by high-ability students (2.77 ± 0.73). The experimental group scored higher on measures of students’ understanding of the content taught (4.46 ± 0.52) (*t* = 4.914, *p* < 0.001), designing good questions for students (4.62 ± 0.51) (*t* = 7.473, *p* < 0.001), and high ability students faced higher level challenges (4.46 ± 0.66) (*t* = 6.223, *p* < 0.001). Finally, in terms of classroom management, the experimental group scored better than the control group in terms of explicit expectations of student behavior (4.31 ± 0.63) and establishing good classroom management connections with each group of students (4.38 ± 0.65) than in terms of explicit expectations of student behavior (3.00 ± 0.82) (*t* = 4.571, *p* < 0.001) and establishing good classroom management connections with each group of students (2.77 ± 0.73) (*t* = 5.980, *p* < 0.001).

**Table 4:** Results of the TSES questionnaire ( $\bar{x}$ ± s).

Investigate the project	Experimental group	Control group	*t*	*P*
Student management				
Make the difficult students obedient	4.08 ± 0.67	3.92 ± 0.64	0.612	0.547
Help students think critically	4.08 ± 0.51	3.23 ± 0.44	4.469	<0.001
Encourage students who are not interested in learning	3.92 ± 0.64	3.77 ± 0.83	0.528	0.602
Let students believe that they can do better in their studies	4.08 ± 0.64	4.00 ± 0.91	0.249	0.806
Helping Students Learn Values	3.54 ± 0.97	3.08 ± 0.64	1.434	0.164
Cultivate students’ creativity	4.23 ± 0.60	3.15 ± 0.69	4.254	<0.001
Teaching strategy implementation				
Answer the difficult questions raised by the students	4.08 ± 0.64	3.92 ± 0.76	0.558	0.582
Measure students’ understanding of what is being taught	4.46 ± 0.52	3.46 ± 0.52	4.914	<0.001
Design good questions for students	4.62 ± 0.51	2.92 ± 0.64	7.473	<0.001
Use multiple evaluation strategies	3.77 ± 0.93	2.77 ± 0.73	3.064	0.005
Provide an alternative response when the student is confused	3.38 ± 0.51	3.54 ± 0.52	−0.765	0.452
Higher level challenges for high ability students	4.46 ± 0.66	2.77 ± 0.73	6.223	<0.001
Classroom management				
Clear expectations for student behavior	4.31 ± 0.63	3.00 ± 0.82	4.571	<0.001
Establish rules to ensure smooth teaching	3.77 ± 0.73	3.38 ± 0.51	1.568	0.130
Let the students obey the rules of the class	4.00 ± 0.71	4.00 ± 0.71	0.000	1.000
Establish good classroom management links with each group of students	4.38 ± 0.65	2.77 ± 0.73	5.980	<0.001
Respond to emergent problems in student learning	3.92 ± 0.64	3.54 ± 0.78	1.378	0.181

## 4 Discussion

Recent research findings highlight that a teaching model, which is supported by intentional learning plans and structured into three distinct phases, significantly enhances students’ learning capabilities, interest, and the overall effectiveness of teachers’ instructional methods. Additionally, our study is same as Xu’s and Wang’s research demonstrates that implementing and evaluating learning guided by well-designed learning plans can enhance students’ autonomous learning ability and foster positive attitudes toward learning (Xu, 2009; Wang, 2015). Implementing instruction based on personalized learning plans not only redefines the conventional roles of teachers and students but also enhances the professional competencies and pedagogical methodologies of teaching faculty (Chen et al., 2024). Consequently, this integration of the three-action teaching model with a guided approach to learning plans significantly enhances students’ enthusiasm and proactive engagement in their educational pursuits, demonstrating the profound impact on both student engagement and educational outcomes.

The implementation of a learning model guided by customized learning plans and structured into three sequential phases within the preventive medicine curriculum of the college has yielded significantly positive outcomes. Evaluation of this innovative teaching strategy, through comparative student testing, revealed that cohorts engaged with this novel approach achieved higher final grades compared to their counterparts in a control group, demonstrating a significant impact on academic performance. Ren (2012) and Gao (2011) reported similar findings. Furthermore, the analysis revealed a consistent upward trend in the disparity of test scores among different experimental groups, indicating a uniform advancement attributed to the model. This pedagogical shift empowers students with increased autonomy, transitioning them from passive recipients of information, as seen in traditional teaching methods, to active and engaged learners. Dunbar also points out that student-centered teaching has achieved a great deal in terms of improving student achievement (Dunbar and Yadav, 2022). Moreover, this approach not only fosters independence but also enhances the interaction between students and educators. The incorporation of thought-provoking inquiries by educators specifically fosters a more profound engagement with the content and facilitates a deeper comprehension of the subject matter. This instructional model encourages active participation and peer-to-peer instruction through classroom activities, thereby enhancing the overall learning experience and educational environment.

The results obtained from the questionnaire on Students’ Socio-Economic Status (SES) demonstrate a significant enhancement in students’ learning experiences through the implementation of structured learning plan guidance and the teaching methodology known as “the three action teaching model” in the preventive medicine course. This approach has notably improved various aspects of learning, including methodological approach, emotional engagement, participation, performance, and overall scores. The experimental group exhibited significantly higher learning methods and total scores compared to the control group. By ensuring a dynamic and effective interaction between teachers and students, enriching the learning content, and promoting knowledge retention post-class, this holistic approach substantively enhances educational practices within the field of preventive medicine while providing a more engaging and comprehensive learning experience. Both our research and Wang’s have shown that this teaching method can improve students’ interest in the subject, so that they can learn to study independently. However, the difference is that Wang’s research shows that students have a low degree of self-preparation completion, less active participation in class, and less review after class (Wang, 2022).

The results obtained from the administration of the Biggs questionnaire within the medical curriculum context provide significant insights into the efficacy of innovative teaching methodologies. Specifically, employing the three-action teaching model with learning plan guidance approach has been associated with a notable increase in students’ motivation scores for deep learning compared to those in a control group. This indicates that students who actively engage with these methods are more inclined to independently explore and comprehend complex concepts, like Lin’s research (Lin, 2015), this teaching model increases student participation in the classroom, which improves overall classroom efficiency. In addition, these students demonstrated excellent deep learning strategies and motivation, indicating that the impact of this approach on students down the road is important because enhancing motivation for learning activities has been shown to promote student engagement and academic performance (Zhao, 2022).

The other SES questionnaire focuses on teacher management and the feasibility of teaching. Consistent with Li et al.’s research findings, teachers can gain a clearer understanding of students’ learning situations and clarify the focal points of class lectures through their knowledge of students’ learning plans (Li, 2011), greatly improve the pertinence of teachers in teaching (Wang, 2022). Overall, the experimental group outperformed the control group in three aspects: student management, teaching implementation, and classroom management. These results demonstrate that when learning is guided by well-designed plans, teachers are better prepared for class and able to differentiate between primary and secondary knowledge.

The success of the new teaching models, which are student-centered and emphasize a customized learning experience, lies in their adaptability and interconnectedness. By prioritizing students’ learning journeys, these models not only facilitate active student participation in their education but also empower them to effectively tackle challenges, thereby reinforcing the fundamental role of “learning.” Additionally, the learning plans excel at identifying and addressing gaps within the teaching framework while reflecting the course’s evolving concepts through well-constructed designs. Ultimately, the adoption of learning plans and the implementation of the “three movements” teaching strategy significantly contribute to enhancing students’ problem-solving capabilities and professional competencies, marking a departure from traditional teaching methodologies toward a more dynamic and participatory learning environment.

## 5 Limitations

The reliability of our survey findings may be compromised due to the limited number of respondents and the brief duration of the survey’s implementation. Additionally, the innovative nature of the model under investigation, which lacks precedent, could introduce significant variability into the results. Simultaneously, it is important to acknowledge that confounding factors may have an influence on this experiment. For instance, students’ varying levels of acceptance toward new concepts, diverse degrees of interest in medical learning, and distinct personal growth experiences should be taken into account. To enhance the robustness of our research and establish a solid foundation for both theoretical understanding and practical application of the three action teaching model with learning plan guidance, it is imperative to expand the scope of our survey, diversify examined courses, enrich content delivery methods, and refine teaching methodologies. This comprehensive strategy will enable us to continuously improve our approach while ensuring its theoretical validity and practical applicability.

## 6 Conclusion

In this study, our objective was to investigate the effects of a novel learning model that integrates the three-action teaching model with learning plan guidance within a preventive medicine course. To achieve this goal, we employed a randomized controlled trial methodology, dividing participants into an experimental group that received the innovative teaching strategy and a control group that experienced traditional teaching methods. Further research on learning outcomes was conducted through surveys, including the Self-Efficacy Scale (SES) and the Biggs survey, which assesses learning approaches. Analysis of the survey data revealed that the experimental group (79.44 ± 10.13) not only achieved significantly higher overall scores compared to the control group (70.00 ± 13.57) (*t* = 3.943, *p* < 0.001), but also demonstrated superior deep learning capabilities as indicated by the results of the Biggs survey. Specifically, the total score for deep learning in the experimental group (28.94 ± 2.57) significantly exceeded that of the control group (25.83 ± 2.38) (*t* = 6.100, *p* < 0.001). The results highlight the efficacy of the proposed model in enhancing both knowledge acquisition and engagement in deeper learning strategies within the context of preventive medicine coursework. The teaching approach of “learning plan guidance” has made a significant breakthrough in imparting concepts and methods, transitioning from passive reception to active mastery, reflecting active construction; from mechanical knowledge replication to developmental mastery and learning, reflecting progress; from rigid teaching procedures to personalized independent learning, emphasizing student autonomy. In summary, the research prospects for “learning plan guidance” remain extensive, necessitating a continued and meticulous study approach. It is imperative to draw insights from frontline teaching experiences and fully explore the immense potential of “learning plan guidance,” enabling it to effectively adapt to the ongoing reforms in modern education. This will ultimately contribute to a substantial enhancement in overall teaching quality (Zhong and Lei, 2009).

## Data Availability

The original contributions presented in the study are included in the article/Supplementary material, further inquiries can be directed to the corresponding authors.
