# Supplementary material for: Implementation and evaluation of the three action teaching model with learning plan guidance in preventive medicine course
Source: Front Psychol. 2024 Nov 7;15:1508432. doi: 10.3389/fpsyg.2024.1508432 (PMC11578742; doi:10.3389/fpsyg.2024.1508432)
Supplement: Supplementary file 1 [file Table_1.DOCX]

Supplementary Material

# Supplementary Figures and Tables

**Supplementary Table1.** Student SES and Biggs questionnaire results（‾*x ± s*）

| Questionnaire | Project | Experimental group | Control group | *t* | *P* |
| --- | --- | --- | --- | --- | --- |
| SES | Learning Methods | 21.72±2.82 | 18.50±2.78 | 5.617 | ＜0.001 |
|  | Learn emotions | 18.60±2.90 | 17.23±2.61 | 2.414 | 0.018 |
|  | Learn to engage | 21.26±3.54 | 19.10±3.36 | 3.041 | 0.003 |
|  | Academic performance | 7.53±1.28 | 6.40±1.18 | 4.493 | ＜0.001 |
|  | Total study score | 69.11±8.39 | 61.23±6.59 | 5.095 | ＜0.001 |
| Biggs | Deep learning motivation | 14.40±1.58 | 11.94±1.72 | 7.272 | ＜0.001 |
|  | Deep learning strategies | 14.53±1.79 | 13.90±1.57 | 1.839 | 0.069 |
|  | Overall score for deep learning | 28.94±2.57 | 25.83±2.38 | 6.100 | ＜0.001 |
|  | Shallow learning motivation | 12.70±2.01 | 13.98±1.96 | -3.134 | 0.002 |
|  | Shallow learning strategies | 12.57±1.82 | 13.65±1.85 | -2.852 | 0.005 |
|  | Total score for shallow learning | 25.26±2.98 | 27.63±3.07 | -3.783 | ＜0.001 |

**Supplementary Figure 1.** Comparison of total scores between test group and control group (points)

**Supplementary Table 2.** Comparison of the total scores of the test group and the control group（‾*x ± s*）

| Constituencies | total scores | Highest score | Lowest score |
| --- | --- | --- | --- |
| Test group  Control group | 79.44±10.13  70.00±13.57 | 94.00  91.00 | 48.00  43.00 |
| *t* | 3.943 |  |  |
| *P* | ＜0.001 |  |  |
